# Overcoming HRP/TMB/H_2_O_2_ Limitations in LFIAs Using Cerium Oxide Nanozymes with Built-In Peroxidase Activity

**DOI:** 10.3390/bios16020096

**Published:** 2026-02-03

**Authors:** John H. T. Luong

**Affiliations:** School of Chemistry, University College Cork, T12 YN60 Cork, Ireland; luongprof@gmail.com

**Keywords:** cerium oxide nanozymes, CeO_2_, HRP replacement, TMB oxidation, lateral flow immunoassay (LFIA), peroxidase-like activity, Ce^3+^/Ce^4+^ redox cycling

## Abstract

Cerium oxide (CeO_2_) nanozymes, also known as nanoceria have emerged as a versatile class of catalytic nanomaterials capable of mimicking key redox enzymes, including oxidases and peroxidases. Their tunable Ce^3+^/Ce^4+^ redox cycling, high density of oxygen vacancies, and exceptional resistance to thermal, pH, and storage stress distinguish CeO_2_ from conventional enzyme labels, such as horseradish peroxidase (HRP). In immunoassays, CeO_2_ enables H_2_O_2_-free TMB (3,3′,5,5′-tetramethylbenzidine) oxidation, generating strong chromogenic signals with minimal background. Although CeO_2_ nanozymes have been explored in colorimetric, chemiluminescent, and photoactive immunoassays, their integration into lateral flow immunoassays (LFIAs) remains limited, with only a few hybrid CeO_2_-containing systems reported to date. This mini-review highlights the limitations of conventional peroxidase-based formats and explains how CeO_2_’s redox cycling (Ce^3+^/Ce^4+^) and oxygen-vacancy-driven catalysis deliver stable, reagent-free signal amplification. Emphasis is placed on the synthetic control of CeO_2_, conjugation chemistry with antibodies, and integration into LFIA architectures. CeO_2_ enables hydrogen-peroxide-free colorimetric detection with improved robustness and sensitivity, positioning it as a promising catalytic label for point-of-care testing. However, it may aggregate in high-ionic-strength buffers, and its synthesis cost increases for highly uniform, vacancy-engineered materials. Surface functionalization with polymers or dopants and optimized dispersion strategies can mitigate these issues, guiding future practical implementations.

## 1. Introduction

Lateral flow immunoassays (LFIAs) are widely used point-of-care tools that rely on capillary-driven migration of labeled antibodies across nitrocellulose membranes. Although inexpensive and simple, most commercial LFIAs use inert gold nanoparticles that provide limited sensitivity because the color intensity depends solely on the number of captured particles. Enzyme-amplified labels, such as horseradish peroxidase (HRP) can enhance signals but are difficult to integrate into dry-reagent formats due to enzyme and hydrogen-peroxide instability. To overcome these constraints, artificial enzyme mimetics—termed nanozymes—have been explored. Among them, cerium oxide (CeO_2_) stands out for its reversible Ce^3+^/Ce^4+^ redox cycle, high oxygen-vacancy density, and robustness under pH and temperature extremes.

Horseradish peroxidase (HRP) coupled with the chromogenic substrate 3,3′,5,5′-tetramethylbenzidine (TMB) and hydrogen peroxide (H_2_O_2_) remains the workhorse signal-generation system in enzyme-linked immunosorbent assays (ELISAs). HRP is inexpensive, readily conjugated to antibodies, and capable of oxidizing a broad spectrum of organic substrates in the presence of H_2_O_2_. TMB has become the preferred chromogen because it provides high molar absorptivity and low background in aqueous buffers [1]. In microplate immunoassays, fresh or refrigerated solutions of HRP–antibody conjugates, TMB, and stabilized H_2_O_2_ are dispensed with precise timing and washing steps, enabling highly sensitive and quantitative detection of proteins, pathogens, and biomarkers across clinical diagnostics, food analysis, and environmental monitoring [2]. However, this classical HRP/TMB/H_2_O_2_ system is not ideally suited for fully integrated lateral flow immunoassays (LFIAs), which are expected to operate as self-contained point-of-care devices. In the strip formats, all reagents must be stored in the dry state, automatically rehydrated by capillary flow, and remain stable for months at ambient or fluctuating temperatures. HRP is susceptible to deactivation during drying and storage, and its catalytic activity decreases upon repeated exposure to H_2_O_2_. Detailed kinetic studies have shown that excess H_2_O_2_ promotes irreversible inactivation of HRP, which has long limited its broader industrial and diagnostic use [3]. Similarly, H_2_O_2_ solutions are chemically unstable, require refrigeration or special stabilization, and are not trivial to integrate as a dry, long-life oxidant on paper substrates [4]. Several groups have developed sophisticated formulations and matrices to enable the long-term dry storage of HRP conjugates and peroxidase substrates for paper-based assays, but these approaches still involve additional manufacturing steps and only partially overcome the intrinsic fragility of HRP and H_2_O_2_ [5]. The need to deliver both TMB and H_2_O_2_ to the test line also adds complexity to the LFIA architecture and compromises the simplicity and robustness that make LFIAs attractive.

Consequently, most LFIAs use colloidal gold nanoparticles (AuNPs) as inert, plasmonic labels that generate a red line without any added substrate. While AuNPs are chemically robust and easy to manufacture, the signal they produce is strictly proportional to the number of particles captured at the test line and is not catalytically amplified. Numerous studies have highlighted the resulting limitations in the analytical sensitivity and quantitative dynamic range of AuNP-based LFIAs, especially compared with enzyme-amplified microplate immunoassays [6]. These constraints have stimulated intense interest in nanozymes—nanomaterials that mimic natural enzyme activities, as next-generation LFIA labels [7].

Among the different nanozyme families, rare-earth oxides (REOs), particularly nanoceria (CeO_2_), are attractive for immunoassay applications. Polymer-coated CeO_2_ nanoparticles possess strong oxidase-like activity at acidic pH, rapidly oxidizing TMB and related chromogens without H_2_O_2_. These nanoparticles could be readily functionalized with targeting ligands for use in ELISA-type immunoassays [8]. Subsequent work demonstrated that nanoceria exhibits multiple redox enzyme-mimicking activities (peroxidase, oxidase, and catalase), governed by its Ce^3+^/Ce^4+^ surface ratio and oxygen vacancies, providing a robust inorganic catalyst that tolerates drying, elevated temperatures, and a broad pH range far better than HRP [9]. When integrated into LFIA formats, these intrinsic activities enable powerful signal amplification directly on the strip. A representative example is the nanoceria-based LFIA for C-reactive protein (CRP), which exploits the oxidase-like activity of CeO_2_ to oxidize TMB without any added H_2_O_2_, generating intense blue test and control lines within 3 min and achieving ng/mL-level detection in human serum [6]. Au@CeO_2_ core–shell nanozymes have been used as LFIA labels for heart-type fatty acid binding protein (H-FABP), where the peroxidase-like activity of the CeO_2_ shell triggers TMB oxidation with a limit of detection down to ~0.35 ng/mL, significantly outperforming analogous AuNP-based assays [10]. These studies collectively indicate that REO nanozymes, particularly CeO_2_ with intrinsic oxidase/peroxidase-like activity, can address several critical limitations of the traditional HRP/TMB/H_2_O_2_ system in lateral flow formats. By eliminating the need for unstable H_2_O_2_, reducing dependence on fragile protein enzymes, and introducing catalytic signal amplification directly at the test line, REO-based nanozymes offer a promising route to LFIAs with markedly improved detection sensitivity and robustness compared with conventional AuNP labels. This mini review highlights the contribution of CeO_2_ nanoparticles in immunoassays to simplify the assay procedure with enhanced detection sensitivity. Their applications in lateral flow immunoassays (LFIAs) are discussed to highlight the potential deployment of this nanozyme.

## 2. Synthesis and Mechanistic Basis of CeO_2_ Peroxidase-like Activity

### 2.1. Synthesis of CeO_2_ NPs

Each synthesis route offers distinct trade-offs: hydrothermal methods provide fine control of size and Ce^3+^/Ce^4+^ ratio but require autoclave conditions; co-precipitation is economical and scalable but prone to agglomeration; sol–gel methods ensure homogeneity yet require long processing times. Plant-mediated synthesis introduces eco-friendliness but limited reproducibility, whereas laser ablation yields pure, ligand-free nanoparticles ideal for biomedical safety testing. Several REO nanoparticles, particularly CeO_2_, Nd_2_O_3_, Sm_2_O_3_, Tb_4_O_7_ and La_2_O_3_, are produced by different methods: hydrothermal, co-precipitation, thermal decomposition, sol–gel routes, and laser ablation (Table 1).

In brief, hydrothermal/solvothermal synthesis is the most frequently encountered method, especially in green synthesis and controlled crystallinity studies. The method produces small, monodisperse nanoparticles (5–20 nm) at low temperature (120–200 °C) and can tune Ce^3+^/Ce^4+^ ratio by changing the reducing agents. Precipitation/co-precipitation is the simplest and most industrially scalable method because it offers a high yield and good batch reproducibility using simple nitrates + NaOH/urea. The thermal decomposition/calcination of the precursors is often combined with precipitation to produce highly crystalline oxides such as CeO_2_, Tb_4_O_7_, Sm_2_O_3_, and Gd_2_O_3_ with controlled grain size via temperature at 500–900 °C. The sol–gel classical method produces uniform, nanocrystalline REO powders with excellent control of the composition. It produces 5–10 nm CeO_2_ to match many nanozyme studies. Green/plant-mediated synthesis was conducted under mild conditions using plant extracts as chelators and reducers. It is useful for the synthesis of antibacterial REOs. Laser ablation in liquid is less common but produces highly pure, ligand-free nanoparticles without chemical residues, which are good for toxicology studies.

### 2.2. Kinetics Compared to HRP

CeO_2_ nanozymes exhibit Michaelis–Menten-type behavior. HRP shows higher catalytic efficiency; however, CeO_2_ nanozymes outperform in terms of stability, cost, reusability, and tolerance to pH/temperature. CeO_2_ NPs act as nanozymes due to the dynamic redox cycle between Ce^3+^/Ce^4+^ and oxygen vacancies. Particularly in the TMB/H_2_O_2_ assays, CeO_2_ can replace HRP to catalyze the two-electron oxidation of TMB to the blue charge-transfer complex ox-TMB (λ_max_ ≈ 652 nm). The overall reaction parallels HRP but does not require a protein structure, making CeO_2_ highly stable under pH, heat, and storage conditions where HRP denatures. The HRP kinetics toward TMB has been well documented with a K_m_ value ranging from 0.04–0.06 mM [17], compared with K_m_ ≈ 0.098 mM of polymer coated CeO_2_ NPs [8], K_m_ = 0.12 mM of citrate-stabilized CeO_2_ nanozyme [14], and K_m_ = 0.032 mM of manganese-doped CeO_2_, (Mn@CeO_2_) [18].

### 2.3. Mechanistic Basis of CeO_2_ Peroxidase-like Activity

CeO_2_ NPs exhibit intrinsic oxidase- and peroxidase-like activities because of their unique mixed-valence Ce^3+^/Ce^4+^ redox couple and abundant oxygen vacancies on the nanoparticle surface. These defects enable rapid electron transfer and reversible cycling between Ce^3+^ and Ce^4+^, a property not found in conventional metal oxides. In aqueous systems, CeO_2_ dynamically modulates its oxidation state according to local redox conditions, allowing it to mimic several natural enzymes, including peroxidase, catalase, and superoxide dismutase. Polymer-coated CeO_2_ NPs catalyze the oxidation of TMB with a strong chromogenic output even in the absence of H_2_O_2_ [8].

### 2.4. CeO_2_/TMB vs. HRP/TMB/H_2_O_2_

Unlike HRP, whose heme center is easily inactivated by excess H_2_O_2_, CeO_2_ remains catalytically competent even at high oxidant concentrations due to its ability to regenerate surface oxygen vacancies. This distinction gives CeO_2_ a clear advantage in highly oxidative or acidic microenvironments where HRP rapidly loses its structural integrity. The relationship between Ce^3+^ concentration and catalytic rate was quantitatively confirmed by Baldim et al. [19]; CeO_2_ nanozyme-based LFIAs offer several practical and mechanistic advantages as summarized below:

(i) The HRP-based system is sensitive to pH, temperature fluctuations, and drying conditions, which limits long-term stability and typically necessitates cold-chain storage. In contrast, CeO_2_ nanozymes remain catalytically active across a broad range of environmental conditions and can be stored at room temperature for extended periods without loss of activity.

(ii) Signal amplification in HRP-based assays requires the external addition of H_2_O_2_, introducing handling complexity and safety concerns. CeO_2_ nanozymes exhibit intrinsic oxidase/peroxidase-like activity and can operate under H_2_O_2_-free conditions, enabling simpler assay formats and improved operational safety. Because of sustained catalytic turnover, CeO_2_-based LFIAs generally achieve higher detection sensitivity (typically in the pg–ng mL^−1^ range) than HRP-based formats (ng/µg mL).

(iii) Although the raw material cost of CeO_2_ nanozymes may be moderately higher than that of HRP, the overall cost per assay becomes comparable when improved shelf life, reduced reagent consumption, and simplified storage requirements are considered. In addition, CeO_2_ nanozyme systems enable faster signal development, typically within 3–5 min, whereas HRP/TMB/H_2_O_2_ assays usually require 10–20 min.

Collectively, these features make CeO_2_ nanozyme-based platforms particularly attractive for robust point-of-care and field-deployable diagnostics.

## 3. Conjugation of CeO_2_ to Detecting Ab (Antibody)

**Figure 1:**
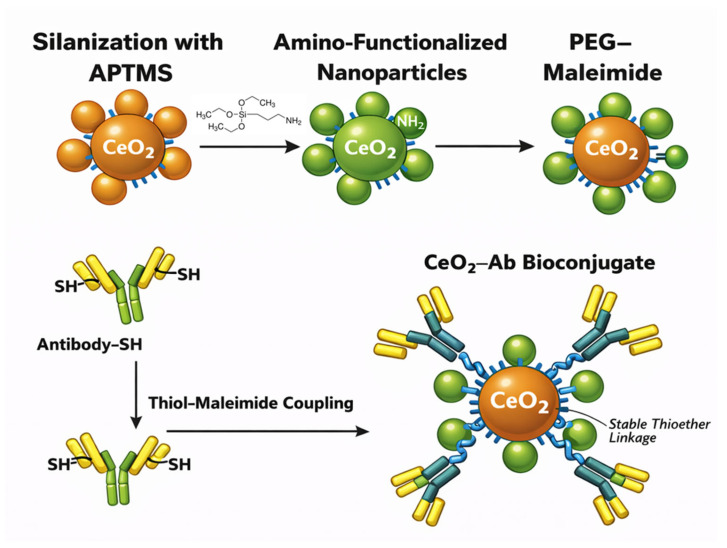
Schematic illustration of the stepwise preparation of CeO_2_–antibody (CeO_2_–Ab) bioconjugates. Nanoceria is first silanized with APTMS to introduce surface amine groups (CeO_2_–NH_2_). The amino-functionalized particles are then reacted with NHS–PEG–maleimide to yield PEGylated CeO_2_ bearing maleimide termini. In parallel, antibodies are thiolated to generate free –SH groups. The thiolated antibodies undergo a maleimide–thiol coupling reaction with PEG–maleimide functional groups on the nanoparticle surface, forming stable thioether linkages and producing the final CeO_2_–Ab bioconjugate.

As an inorganic support, CeO_2_ nanoparticles possess abundant surface –OH and lattice O^2−^ sites, rendering them hydrophilic yet prone to aggregation. To enhance stability and provide reactive groups, CeO_2_ NPs are commonly coated with organic layers bearing –COOH or –NH_2_ functionalities, enabling standard EDC/NHS coupling for antibody (Ab) immobilization. PEG–NHS-functionalized CeO_2_ (Mal-PEG-NHS–CeO_2_) can be synthesized following procedures established for iron oxide nanomaterials [20,21]. Typically, CeO_2_ is first modified with a silane coupling agent such as (3-aminopropyl)trimethoxysilane (APTMS) to introduce surface amines. Thiolated antibodies are then linked through a heterobifunctional NHS–PEG–maleimide spacer, yielding oriented, site-specific conjugation [22] (Figure 1). Alternatively, polymer coatings such as chitosan provide amino-terminated CeO_2_–NH_2_ conjugates for covalent Ab attachment [23]. Although glutaraldehyde can crosslink amine-functionalized CeO_2_ and antibodies, its non-selective reactivity may impair antigen-binding sites; hence, maleimide–thiol coupling is generally preferred.

A recent study showed how particle size and surface chemistry affect CeO_2_’s catalytic activity. Therefore, it is important to optimize these two features of CeO_2_ before linking to proteins [14]. The size-dependent catalytic behavior of CeO_2_ nanozymes and their stability under harsh conditions (pH 2–10, up to ~200 °C, and long-term storage) have been extensively investigated [14]. Overall, the oxidation of TMB by CeO_2_ closely parallels the HRP catalytic cycle but does not require a protein scaffold, heme cofactor, or a narrowly defined pH/temperature window. This makes CeO_2_ exceptionally robust in biosensing systems, such as biosensors, paper-based devices, and LFIAs, where protein enzymes (HRP) often denature upon drying, undergo peroxide-mediated inactivation, or require strict storage conditions. As a result, CeO_2_ nanozymes offer a powerful alternative catalytic label capable of producing strong, stable, and reproducible chromogenic signals for advanced immunoassays.

**Figure 2:**
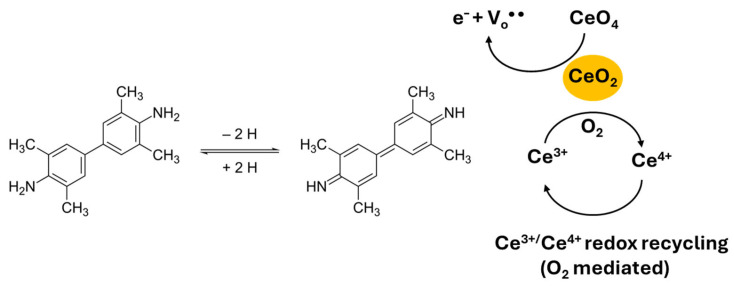
CeO_2_ mimics peroxidase activity through the reversible redox cycling of surface Ce^4+^/Ce^3+^ ions and oxygen vacancies (V_0_^••^). Ce^4+^ oxidizes TMB to ox-TMB, while Ce^3+^ is regenerated by reaction with dissolved oxygen (O_2_) or H_2_O_2_, completing a catalytic loop.

A mechanistic of the CeO_2_-catalyzed TMB oxidation is shown in Figure 2. CeO_2_ mimic peroxidase activity through the reversible redox cycling of surface Ce^4+^/Ce^3+^ ions and oxygen vacancies (V_0_^••^). Ce^4+^ oxidizes TMB to ox-TMB (λ_max_ ≈ 652 nm), while Ce^3+^ is regenerated by reaction with dissolved oxygen, completing a catalytic loop analogous to HRP but without the need for a protein structure. This inorganic mechanism confers exceptional stability to CeO_2_ under harsh pH, temperature, and storage conditions, making CeO_2_ nanozymes highly attractive as catalytic labels in immunoassays and LFIAs.

## 4. LFIAs Equipped with CEO_2_ Nanozyme

**Figure 3:**
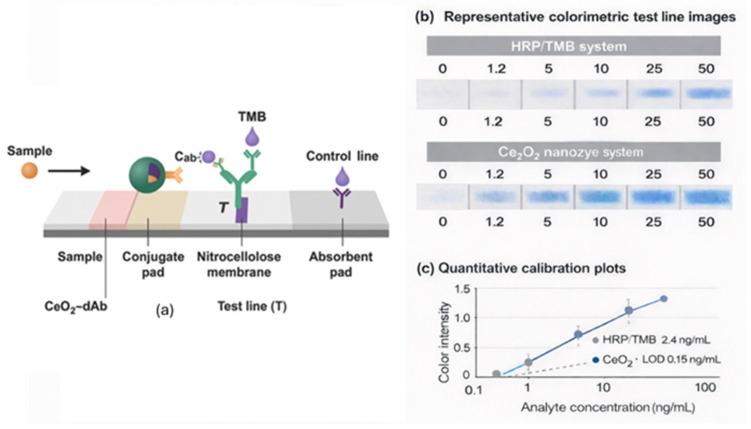
(**a**) Schematic illustration of the CeO_2_–antibody (CeO_2_–dAb)-labeled LFIA, showing the sample flow through the conjugate pad, nitrocellulose membrane, and absorbent pad, with color development at the test line (T) due to CeO_2_-catalyzed TMB oxidation. (**b**) Representative colorimetric test line images comparing the HRP/TMB/H_2_O_2_ system and the CeO_2_ nanozyme system at increasing analyte concentrations, demonstrating enhanced signal intensity and stability for CeO_2_. (**c**) Quantitative calibration plots of color intensity versus analyte concentration showing higher sensitivity and a lower detection limit (LOD ≈ 0.15 ng mL^−1^) for CeO_2_-based LFIAs compared with the HRP system (LOD ≈ 2.4 ng mL^−1^). Panels (**b**,**c**) were redrawn and modified based on representative data from Kong et al. [6].

A schematic illustration of a CeO_2_ nanoparticle–enhanced lateral flow immunoassay (LFIA) is shown in Figure 3a. After sample introduction, the analyte binds to CeO_2_-labeled detection antibodies (CeO_2_–dAb) released from the conjugate pad. During capillary migration, the complex is captured at the test line (T) by immobilized capture antibodies (cAb), forming a ternary sandwich complex (cAb–analyte–dAb–CeO_2_). TMB is deposited directly onto both the testing (T) and control (C) lines, where the intrinsic oxidase-like catalytic activity of CeO_2_ produces a localized blue color. The intensity of the test line reflects the analyte concentration, whereas the control line serves as a procedural control. Excess CeO_2_–dAb is retained at the control line (C) by anti-IgG antibodies, confirming proper flow and reagent activity.

Unlike AuNPs, which generate passive red signals via localized surface plasmon resonance, CeO_2_ NPs act as enzyme mimetics, catalyzing the oxidase-like conversion of TMB into an intense blue chromophore. Each CeO_2_ particle can oxidize thousands of TMB molecules, yielding post-binding signal amplification at the test line. This catalytic amplification overcomes the primary sensitivity limitation of AuNP-based LFIAs, where the signal intensity is strictly dependent on the number of nanoparticles captured by the immobilized antibodies. Moreover, CeO_2_ nanozymes are highly stable under dry storage, humidity, and thermal stress, conditions under which HRP loses its activity. Their robustness enables integration into low-cost LFIA strips while maintaining excellent analytical performance.

Despite extensive research on CeO_2_ nanozymes, their integration into lateral flow immunoassays (LFIAs) remains extremely limited. To date, only one CeO_2_-related LFIA system has been reported: an Au@CeO_2_ hybrid nanozyme LFIA for heart-type fatty acid binding protein (H-FABP) [10], an early biomarker of myocardial injury. In this design, a gold core provides strong optical contrast while the CeO_2_ shell contributes oxidase-like catalytic amplification, enabling a sandwich LFIA with a detection limit of ~0.35 ng/mL—sufficient for early clinical triage. This example demonstrates that CeO_2_-based nanozymes can be successfully incorporated into LFIA architectures and can substantially enhance analytical performance, yet it also highlights how underexplored this area remains, with no published CeO_2_-based LFIAs for other major biomarkers such as cardiac troponin I, viral antigens, or food toxins.

Nonetheless, the successful use of Au/CeO_2_ nanoparticles in highly sensitive optical fiber sensors for cardiac troponin I [24] suggests a clear translational opportunity. Extending CeO_2_-based nanozymes into LFIA formats for cTnI and other clinically relevant biomarkers—such as CRP or SARS-CoV-2 nucleocapsid protein—represents a logical and technically feasible next step, particularly given the strong oxidase-like activity, stability, and low-cost synthesis of CeO_2_. Another closely related example that reinforces the suitability of CeO_2_ nanozymes for point-of-care diagnostics—although not a lateral flow strip—is the two-dimensional dissipative paper-based immunoassay developed by Yu et al. [25] for portable cancer screening. In this system, oxygen-vacancy-enriched CeO_2_ nanosheets were engineered to expose catalytically active (100) facets, enabling strong oxidase-like turnover of TMB directly on a cellulose substrate. While the assay does not rely on capillary-driven flow as in LFIA, it shares several architectural and functional similarities: it is fully paper-based, uses antibody–nanozyme conjugates, performs chromogenic readout on nitrocellulose-like matrices, and leverages CeO_2_’s catalytic amplification to boost sensitivity without external reagents. These parallels demonstrate that CeO_2_ nanozymes are already compatible with paper substrates, antibody immobilization, and colorimetric signal generation—three of the core design principles of LFIA—suggesting that translating CeO_2_ nanozymes into true lateral flow formats is technically feasible and scientifically underexplored.

A further example demonstrating the diagnostic versatility of CeO_2_ nanozymes is the photoactive CeO_2_/g-C_3_N_4_ heterostructure reported by Cheng et al. [26] for carcinoembryonic antigen (CEA) detection. In this system, CeO_2_ nanoparticles were coupled with graphitic carbon nitride to form a Z-scheme nanozyme with enhanced charge separation and light-driven catalytic activity. Under visible light irradiation, the hybrid nanozyme generated reactive oxygen species capable of oxidizing chromogenic substrates, enabling a sensitive colorimetric immunoassay on a paper-compatible platform. Although this design is not a lateral flow strip, it shares several conceptual features with LFIA-style nanozyme amplification—antibody–nanozyme conjugation, chromogenic readout, and catalytic signal enhancement occurring directly on a solid support. The CeO_2_/g-C_3_N_4_ system therefore reinforces the broader theme that CeO_2_-based nanozymes can be engineered for high-performance biomarker detection across diverse paper-based and solid-phase formats, further supporting their potential translation into future LFIA architectures.

Another recent advance that expands the diagnostic scope of CeO_2_-based nanozymes is the chemiluminescent immunochromatographic assay developed by Luo et al. [27], who engineered CeO_2_@Ru nanospheres through a size–metal-loading synergy strategy. In this hybrid design, CeO_2_ provides a high-surface-area catalytic scaffold while Ru enhances chemiluminescence efficiency, enabling strong signal amplification along the test line of an immunochromatographic strip. Although the mechanism differs from traditional oxidase-like colorimetric amplification, the CeO_2_@Ru system demonstrates that CeO_2_-containing nanozymes can be successfully integrated into LFIA-type architectures and can support highly sensitive detection through catalytic emission. This work further reinforces the versatility of CeO_2_ nanozymes across multiple assay modalities—colorimetric, photoactive, and now chemiluminescent—highlighting their potential for next-generation point-of-care diagnostics.

Although these earlier CeO_2_ nanozyme studies focused on glucose rather than protein biomarkers, they remain highly relevant to the present discussion because they establish the fundamental catalytic behavior that underpins all CeO_2_-based diagnostic platforms. Wu et al. [28] and Wang et al. [29] demonstrated that CeO_2_ nanoparticles possess strong intrinsic oxidase- and peroxidase-like activity capable of driving TMB oxidation on paper substrates without external reagents, providing clear, stable colorimetric signals. These mechanistic insights justify the inclusion of these references: they show that CeO_2_ nanozymes already meet several core requirements for LFIA translation—paper compatibility, reagent-free chromogenic amplification, and robust catalytic turnover. In this sense, the glucose studies are not tangential but foundational, illustrating why CeO_2_ is a promising candidate for future LFIA development and helping to contextualize the more recent biomarker-focused advances.

A comparison of CeO_2_ and Iron-Oxide Nanozymes for TMB oxidation deserves a brief discussion here because iron-oxide nanozymes (Fe_3_O_4_ “IONzymes”) were the first nanomaterials reported to catalyze TMB/H_2_O_2_ oxidation [30]. In brief, engineered CeO_2_ nanozymes can equal or surpass Fe_3_O_4_ in both catalytic efficiency and operational stability [14]. Fe_3_O_4_ exhibits strong peroxidase-like activity and low K_m_ values for TMB, but its performance heavily depends on the acidic conditions (optimal pH ≈ 3–4) and the continuous availability of H_2_O_2_. Furthermore, surface Fe^2+^ sites are gradually oxidized to Fe^3+^ under catalytic turnover, reducing the long-term activity. In contrast, CeO_2_ nanozymes rely on the reversible Ce^3+^/Ce^4+^ redox cycle and oxygen vacancies, enabling them to maintain a sustained catalytic output across a wider pH range, resist oxidative deactivation, and remain stable during drying and storage. Importantly, vacancy-rich CeO_2_–x materials have demonstrated significantly higher catalytic turnover (k_cat_) than Fe_3_O_4_ in TMB assays, with reported enhancements up to an order of magnitude under identical conditions [31]. CeO_2_ also provides an additional advantage through its oxidase-like activity, enabling TMB oxidation even without added H_2_O_2_. This is an important benefit for integration into self-contained diagnostic platforms such as LFIAs. These findings indicate that CeO_2_ nanozymes, particularly oxygen-deficient CeO_2_–x, offer superior robustness and, in many cases, higher catalytic performance than Fe_3_O_4_, making them strong candidates for advanced chromogenic immunoassays and point-of-care diagnostic devices.

## 5. Perspectives

CeO_2_ nanozymes offer several major advantages over HRP in LFIAs. Their catalytic oxidation of TMB provides strong signal amplification, overcoming the inherent sensitivity limits of nanoparticle-based assays and enabling detection down to the low-picogram per milliliter range—well suited for demanding biomarkers such as cTnI, NT-proBNP, procalcitonin, and various cancer markers. They also exhibit exceptional robustness: CeO_2_ retains activity after drying, rehydration, humidity exposure, and long-term storage, making it far more reliable than HRP for point-of-care use, field diagnostics, home testing, and low-resource settings without cold-chain infrastructure. In addition, CeO_2_’s tunability through coatings, dopants, and particle-size engineering supports multiplexed and microfluidic LFIA formats, where catalytic amplification keeps each test line visually distinct even at very low analyte levels. Finally, the intense blue CeO_2_–TMB signal is highly compatible with smartphone imaging and lightweight AI analysis, enabling semi-quantitative or fully quantitative readouts that overcome the limitations of subjective visual interpretation.

Nonetheless, several important developments are still needed for practical applications. These include the creation of preloaded dry TMB pads to enable fully self-contained strips without the need for reagent drops, the integration of CeO_2_–enzyme cascades such as HRP + CeO_2_ to achieve ultralow detection limits, and the strategic doping of cerium oxide with rare-earth elements like samarium, gadolinium, and terbium to fine-tune the Ce^3+^/Ce^4+^ ratio and enhance catalytic efficiency. Additionally, a broader clinical deployment is required for detecting cardiac biomarkers, inflammatory markers, infectious diseases, and environmental monitoring.

Biotin-streptavidin chemistry further amplifies signals in immunoassays by leveraging streptavidin’s four binding sites for biotin, allowing multiple reporter molecules to attach to a single biotinylated target, creating a strong, detectable signal even for low-abundance analytes. However, there has been a growing concern of emerging biotin interference in clinical immunoassays due to the overconsumption of biotin [32,33]. Many commercial immunoanalyzers no longer use this amplification strategy to avoid this serious interference of biotin in the assay samples.

Despite the aforementioned advantages, CeO_2_ nanozymes also present several challenges. Their catalytic activity decreases significantly under strongly basic conditions, consistent with earlier observations that redox behavior and catalytic turnover are highly pH-dependent in nanoceria systems [30]. Aggregation can also occur in high-ionic-strength or protein-rich buffers, reducing mobility on nitrocellulose and limiting catalytic accessibility, a behavior linked to surface-charge-dependent stability and dispersion issues in CeO_2_ nanoparticles [8]. In addition, achieving highly uniform, vacancy-engineered CeO_2_ with controlled Ce^3+^/Ce^4+^ ratios increases synthesis complexity and cost, as defect density and oxygen-vacancy engineering strongly influence catalytic performance. Surface functionalization with polymers or rare-earth dopants such as Gd^3+^ or Tb^3+^ can mitigate aggregation and tune redox properties [9], while optimized dispersion strategies help reduce nonspecific catalytic background, supporting more reliable implementation in practical immunoassays.

## 6. Conclusions

CeO_2_ nanozymes represent a significant advancement in immunodiagnostics by combining enzyme-like catalytic amplification with inorganic robustness unmatched by protein enzymes. Their ability to oxidize TMB in the absence of H_2_O_2_ reduces complexity and enhances biosafety, while their redox-active surfaces enable strong visual signals even at low analyte concentrations. Compared with AuNP labels, which rely solely on passive plasmonic coloration, CeO_2_ provides active catalysis, dramatically improving the analytical sensitivity without compromising the assay stability. The growing body of literature demonstrates that CeO_2_-based immunoassays—both plate-based ELISAs and LFIAs—achieve clinically relevant detection limits for cardiac markers, inflammatory biomarkers, toxins, and viral antigens. As functionalization strategies become more refined (e.g., polyethylene glycol; (PEGylation, polyacrylic acid (PAA)-coating, and silane chemistry), the conjugation of antibodies, enzymes, or other biomolecules onto CeO_2_ nanoparticles has become increasingly reliable and reproducible. Overall, CeO_2_ nanozymes represent a maturing technology with strong practical advantages and clear pathways toward widespread diagnostic deployment. Although CeO_2_ nanozyme-based LFIAs have not yet been demonstrated for viral antigens such as Influenza A NP or SARS-CoV-2 N protein, the catalytic amplification achieved in CeO_2_-enhanced LFIA systems for nonviral biomarkers indicates substantial untapped potential. Future work integrating CeO_2_ nanozymes with viral immunoassays may enable next-generation point-of-care diagnostics with sensitivities approaching molecular methods.

## Figures and Tables

**Table 1:** Widely Used Synthesis Routes for CeO_2_.

Synthesis Method	Typical REOs Produced	Key Features	Reference
Rapid microwave—Hydrothermal synthesis	CeO_2_	Single cubic phase spherical CeO_2_ nanoparticles with average particle size of ∼15 nm.	[11]
Precipitation/Co-precipitation (NaOH, NH_4_OH, urea)	CeO_2_	Simple, scalable, high throughput; precursor hydroxide → oxide via calcination	[12]
Plant-mediated/”Green” Synthesis	CeO_2_	Biomolecules act as reducing & capping agents; low toxicity; eco-friendly	[13]
Sol–Gel Synthesis	CeO_2_ (5–10 nm)	Excellent compositional homogeneity; small particle size; mild conditions	[14]
Calcination/Thermal Decomposition	CeO_2_	Produces highly crystalline REOs; temperature controls grain size (400–900 °C)	[15]
Laser Ablation in Liquid (LAL)	CeO_2_ (ultrapure, ligand-free nanoparticles)	Produces clean, surfactant-free NPs; no byproducts; high stability	[16]
Polymer-Assisted Reduction/Stabilization	CeO_2_ nanozymes (oxidase/peroxidase-like catalysts)	Polymer shells increase Ce^3+^ surface content; improve colloidal stability	[8]
Citrate/Carboxylate-Stabilized Nanosynthesis	CeO_2_	Surface citrate → high colloidal stability; widely used in bio- and nanozyme systems	[9]
